# *Agapetesheana*, a new species of A.ser.Longifiles (Ericaceae) from Yunnan, China

**DOI:** 10.3897/phytokeys.180.69667

**Published:** 2021-08-09

**Authors:** Yi-Hua Tong, Ying Bai, Jian-Ming Feng, Ji-Dong Ya

**Affiliations:** 1 Key Laboratory of Plant Resources Conservation and Sustainable Utilization & Key Laboratory of Digital Botanical Garden of Guangdong Province, South China Botanical Garden, Chinese Academy of Sciences, Guangzhou, 510650, China South China Botanical Garden, Chinese Academy of Sciences Guangzhou China; 2 Center of Conservation Biology, Core Botanical Gardens, Chinese Academy of Sciences, Guangzhou, 510650, China Core Botanical Gardens, Chinese Academy of Sciences Guangzhou China; 3 Forestry and Grassland Bureau of Honghe Prefecture, Honghe Avenue, Mengzi, Yunnan, 661100, China Forestry and Grassland Bureau of Honghe Prefecture Mengzi China; 4 Germplasm Bank of Wild Species, Kunming Institute of Botany, Chinese Academy of Sciences, Lanhei Road 132, Heilongtan, Kunming, Yunnan, 650201, China Kunming Institute of Botany, Chinese Academy of Sciences Kunming China

**Keywords:** China-Vietnam border, epiphytic, Huanglian Shan, morphology

## Abstract

*Agapetesheana* Y. H. Tong & J. D. Ya (Ericaceae), a new species from Lüchun Xian, Yunnan Province, China is described and illustrated. This new species is assigned to Agapetessect.Agapetesser.Longifiles Airy Shaw. It is closest to *A.inopinata* Airy Shaw and *A.oblonga* Craib, but differs in having bead-like tubers, leaf blade with a wholly serrulate margin, subulate and much longer calyx lobes, much larger corollas that are carmine, green at the apex and maroon on angles, and longer stamens without spurs on the back.

## Introduction

A general introduction to *Agapetes* D. Don ex G. Don, focusing on the species in China was given in previous papers published by the first author and is not repeated here (Tong 2016; Tong et al. 2019). With 17 species and two varieties of *Agapetes* including the recently published *A.yingjiangensis* Y. H. Tong, B. M. Wang & N. H. Xia, Yunnan Province, after Tibet, harbors the second most species of this genus in China (Huang and Fang 1991; Fang and Stevens 2005; Tong 2014). During one recent field trip to Huanglian Shan National Nature Reserve, Yunnan Province, China, an unknown *Agapetes* species was discovered. The combination of its ovate to ovate-lanceolate leaf blades, inflorescence with glandular hairs and elongated filaments immediately reminded us of two other similar species from the same province, viz. *A.oblonga* Craib and *A.inopinata* Airy Shaw. However, the latter two species have very differently colored corollas. After examining the specimens of similar species and referring to the related literature (Hiep 2003; Kress et al. 2003; Fang and Stevens 2005; Banik and Sanjappa 2014; Watthana 2015), we concluded that this unknown species is a new one to science, which is described and illustrated below.

## Materials and methods

Specimens were collected from Huanglian Shan National Nature Reserve, Yunnan Province, China during two field expeditions in March and April 2021, respectively. All descriptions were based on dried specimens, which were deposited at the herbaria of Kunming Institute of Botany, Chinese Academy of Sciences (**KUN**) and South China Botanical Garden, Chinese Academy of Sciences (**IBSC**).

## Taxonomic treatment

### Agapetes heana

Taxon classificationPlantaeEricalesEricaceae

Y. H. Tong & J. D. Ya sp. nov.

730AF5FC-372F-5D19-BAC6-8900D8E75516

urn:lsid:ipni.org:names:77218883-1

Figures 1 , 2

#### Type.

China. Yunnan Province: Lüchun Xian, Huanglian Shan National Nature Reserve, elev. 1803 m, 4 March 2021 (fl.), *J. H. He 210304* (***holotype***KUN).

#### Diagnosis.

*Agapetesheana* is similar to *A.inopinata* Airy Shaw and *A.oblonga* Craib in the leaf blade shape, the glandular hairy inflorescence and the filaments that are longer than thecae, but can be distinguished from the latter two by its bead-like tubers (vs. spindle-shaped), leaf blade with a wholly serrulate margin (vs. entire, or inconspicuously serrate beyond middle, or sparsely denticulate at apex), subulate (vs. triangular) and much longer (7–8 mm vs. ca. 1 mm and 1.5–2.0 mm, respectively) calyx lobes, much larger (ca. 3.5 cm vs. ca. 0.8 cm and 1.3–1.9 cm, respectively) corollas that are carmine, green at the apex and maroon on angles (vs. red, crimson or carmine), and longer (ca. 3.6 cm vs. ca. 0.7 cm and 1.0–1.5 cm, respectively) stamens that are without spurs on the back (vs. with 2 obvious short spurs) (Table 1).

**Table 1.** A morphological comparison among *Agapetesheana*, *A.inopinata* and *A.oblonga*.

Characters	* A. heana *	* A. inopinata *	* A. oblonga *
Tubers	Bead-like	Spindle-shaped	Spindle-shaped
Leaf blade margin	Serrulate	Entire	Entire or inconspicuously serrate beyond middle, or sparsely denticulate at apex
Inflorescence	Shortly racemose, 2–3-flowered	Racemose, 4–6-flowered	Fasciculate, 1–4-flowered
Calyx lobes	Subulate, 7–8 mm long	Triangular, ca. 1 mm long	Triangular, 1.5–2.0 mm long
Corolla color	Carmine, green at the apex and maroon on angles	Red	Crimson or carmine
Corolla length	Ca. 3.5 cm	Ca. 0.8 cm	1.3–1.9 cm
Corolla lobes	Narrowly triangular, 6–7 mm long	Triangular, ca. 1 mm long	Triangular, ca. 1.5 mm long
Stamen length	Ca. 3.6 cm	Ca. 0.7 cm	1.0–1.5 cm
Spurs on the back of anthers	Absent	Present	Present

**Figure 1.**
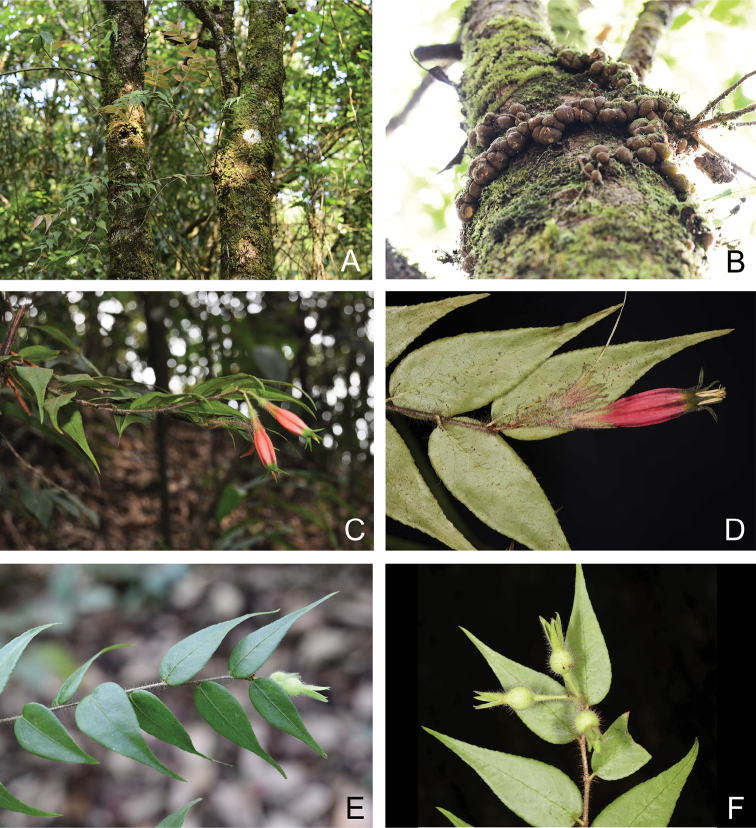
*Agapetesheana***A** habit **B** tubers **C** flowering branch **D** inflorescence **E** fruiting branch **F** fructescence with young fruits (**C, D** from *J. H. He 210304*, **A, B, E, F** from *J. D. Ya et al. 21CS20738*). All photographs by J. D. Ya except **C** by J. H. He.

#### Description.

Evergreen shrub, epiphytic on trees. Tuber globose, 1–3 cm in diam, bead-like. Stems and branches slender, obliquely spreading. Twigs greyish brown, terete, 2–3 mm in diam., densely glandular-setose and pubescent, glabrescent when old. Leaves sub-distichously scattered; petiole 2–3 mm long, pubescent adaxially, glandular-setose and pubescent abaxially; leaf blades thinly leathery or firmly papery, ovate to ovate-lanceolate, 4.5–7.0 × 1.5–2.6 cm, adaxially glabrous except the pubescent midvein, abaxially sparsely glandular-setose, more so on midvein, trichomes deciduous when old, midveins conspicuously raised above, slightly raised below, secondary veins 6–11 pairs, with veinlets conspicuous on both sides, base rounded to slightly cordate, without basal glands, margin slightly revolute when dry, each side with 20–24 serrula, each serrula with a glandular seta at the tip, setae deciduous, apex caudate-acuminate. Inflorescences shortly racemose, 2–3-flowered, 0.3–1.0 cm long, pseudo-terminal, densely glandular-setose and pubescent; bracts unknown; pedicels 9–10 mm long, densely glandular-setose and pubescent, slightly expanded upwards; bracteoles 2, basal, ovate, ca. 1 mm long, brown, deciduous. Calyx tube green, 3.0–3.5 mm long, densely glandular-setose and pubescent, trichomes slightly denser and longer than those on pedicels; limb 1.1–1.2 cm long, densely glandular-setose and pubescent, lobes tinged with carmine, subulate, 7–8 × 1.5–2.0 mm, densely glandular-setose and pubescent outside, pubescent inside, apex acute. Corolla carmine, green at the apex and maroon on angles, tubular, slightly 5-angled, ca. 3.5 × 0.7–0.9 cm, sparsely glandular-setose and pubescent along the upper half of angles outside, glabrous inside; lobes green, spreading, narrowly triangular, 6–7 mm long, glandular-setose and pubescent on midvein outside, nearly glabrous inside. Stamens 10, ca. 3.6 cm long; filaments flat, ca. 2.5 cm long, glabrous; anthers ca. 1.45 cm long, thecae adnate to each other, ca. 4.5 mm long, tubules ca. 1 cm long, without spurs on the back. Style slender, 3.8–4.0 cm long; stigma truncate; ovary 10-pseudoloculed, each locule with several ovules; disk glabrous. Young fruit green, subglobose, densely glandular-setose and pubescent, with erect persistent calyx lobes at apex.

**Figure 2:**
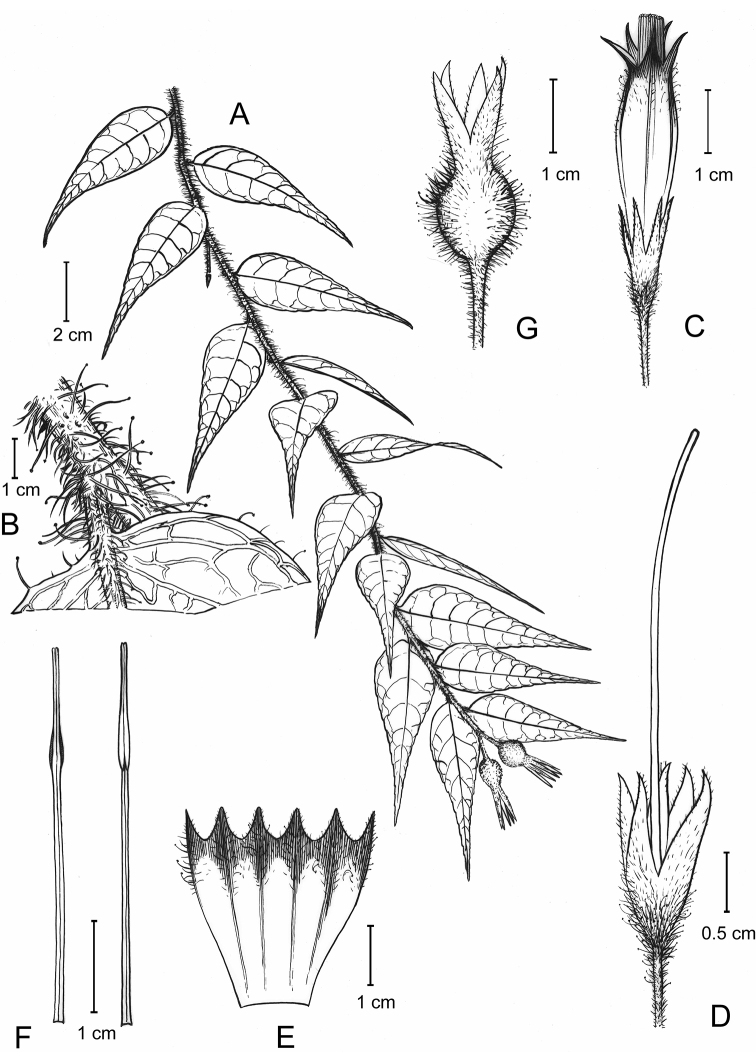
. *Agapetesheana***A** fruiting branch **B** trichomes on branch and petiole **C** flower **D** calyx and style **E** opened corolla, abaxial view **F** stamens, abaxial (left) and adaxial (right) view **G** young fruit (**A, G** from *J. D. Ya et al. 21CS20738*, **B–F** from *J. H. He 210304*). Drawn by Mr. D. H. Cui.

#### Etymology.

The species epithet is named in honor of Ms. Jiang-Hai He, a local staff in Huanglian Shan National Nature Reserve, who has worked there for almost 30 years and made a big contribution to the knowledge of biodiversity of this nature reserve, and is also the discoverer of this new species.

#### Vernacular name.

疆海树萝卜(Chinese pinyin: Jiāng hăi shù luó bo).

#### Distribution and habitat.

This species is currently known only from the type locality, i.e. Huanglian Shan National Nature Reserve, Yunnan, China. Since this locality is very close to the border of China and Vietnam and the habitat is similar and continuous, this species is probably also distributed in Vietnam. It grows on the trunks of trees like *Schimawallichii* (DC.) Korth. or *Lithocarpus* sp. under broadleaved forests at an elevation of ca. 1800 m.

#### Conservation status.

*Agapetesheana* seems to be very rare in the type locality, since only a population of fewer than 10 individuals has been found for now, but the threat risk seems to be low because it is not economically valuable and the conservation condition of the reserve is good. Because no population assessment of this species in the field of China or adjacent area of Vietnam has been made, it is best classified as ‘Data Deficient’ (DD) (IUCN Standards and Petitions Committee 2019).

#### Additional specimens examined.

**(*paratypes*)** The same locality as holotype, 8 April 2021 (young fruits), *J. D. Ya et al. 21CS20738* (KUN, IBSC).

## Discussion

According to Airy Shaw’s infrageneric system, *A.heana* fits well with the circumscription of Agapetessect.Agapetesser.Longifiles Airy Shaw due to its slender stems, flowers arranged into a short raceme, and elongated filaments (longer than anthers) (Airy Shaw 1935, 1959), except that its anthers are not spurred, while almost all the species of that series own spurred anthers. Both spurred and unspurred anthers occur in other series, such as ser. Agapetes and ser. Pteryganthae (Airy Shaw 1935, 1948, 1959; Huang 1991). Thus, this character appears to have evolved more than once in this genus. Besides this new species, there are another two species of *Agapetes* distributed in the same mountain (Huanglian Shan), viz. *A.lobbii* C. B. Clarke (*S. K. Wu et al. 652*, PE!, KUN0230909!) and *A.rubrobracteata* R. C. Fang & S. H. Huang (*S. K. Wu et al. 253*, PE!). However, these two species are distantly related to our new species due to their very different vegetative and productive characters, such as habits, leaf blade shapes, indumentum on branches and inflorescences, various floral characters, and so on.

## Supplementary Material

XML Treatment for Agapetes heana
